# Simple Detection Methods for Senescent Cells: Opportunities and Challenges

**DOI:** 10.3389/fragi.2021.686382

**Published:** 2021-07-06

**Authors:** Richard G. A. Faragher

**Affiliations:** Centre for Stress and Age-Related Disease, School of Applied Sciences, University of Brighton, Brighton, United Kingdom

**Keywords:** ageing, senescence, lipofuscin, labelling index, detection

## Abstract

Cellular senescence, the irreversible growth arrest of cells from conditional renewal populations combined with a radical shift in their phenotype, is a hallmark of ageing in some mammalian species. In the light of this, interest in the detection of senescent cells in different tissues and different species is increasing. However much of the prior work in this area is heavily slanted towards studies conducted in humans and rodents; and in these species most studies concern primary fibroblasts or cancer cell lines rendered senescent through exposure to a variety of stressors. Complex techniques are now available for the detailed analysis of senescence in these systems. But, rather than focussing on these methods this review instead examines techniques for the simple and reproducible detection of senescent cells. Intended primary for the non-specialist who wishes to quickly detect senescent cells in tissues or species which may lack a significant evidence base on the phenomenon it emphasises the power of the original techniques used to demonstrate the senescence of cells, their interrelationship with other markers and their potential to inform on the senescent state in new species and archival specimens.

## What is a Senescent Cell?

### Historical Background

The initiation of the first true cultures of metazoan cells by Alexis Carrel (Carrel, 1913) is a landmark in the history of biology. Carrel was primarily interested in understanding the regulatory factors controlling wound healing. In attempting to do so he established cell culture as a valuable experimental tool for the study of cell morphology and function. Gradual refinement of tissue culture techniques have allowed the culture of progressively more complex cell types and the development of model systems for the study of many fundamental biological processes.

Initially however, studying ageing *in vitro* appeared impossible. Although it had been proposed that exhaustion of the growth capacity of somatic cells was responsible for organismal ageing (Wesimann, 1889), Carrel’s best known experiments appeared to falsify this hypothesis. Carrel initiated cultures of chick fibroblasts which his co-workers claimed to have cultured continuously for over 30 years, much longer than the lifespan of the intact organism (Ebeling, 1942). It followed that the ageing process had to operate at the level of the tissue or organism not the single metazoan cell, which seemed as immortal as its bacterial counterpart. As tissue culture gained in popularity through the 1950s the failure to produce cultures of “immortal” cells was attributed to poor experimental technique. This was not innately implausible given the extraordinary difficulties then involved in carrying out tissue culture experiments that today would be considered trivial. This “poor culture technique” argument has reappeared regularly as cytologists have pushed the boundaries of tissue culture to the technological limits of their day (Perillo et al., 1989) and will doubtless reoccur in the future, sometimes justifiably.

However, in the early 1960’s (Hayflick and Moorehead, 1961; Hayflick, 1965) Hayflick demonstrated that normal human fibroblasts would only proliferate for a finite number of passages in culture. Latent infectious agents, composition of the medium and depletion of key metabolites were all shown not to be responsible for this failure to grow. Hayflick also observed that fibroblast cultures initiated from embryos grew substantially better than those derived from adults. Combined with the demonstration that normal somatic cells displayed limited lifespans during serial transplantation experiments these observations, led Hayflick to formulate *a cellular theory of ageing* (Hayflick, 1965; Hayflick, 1979). The original tenets of this hypothesis were that a finite lifespan is an intrinsic property of normal human cells, that cell growth *in vitro* is somehow related to human ageing and that cultured primary fibroblasts were a useful model system in which to study some aspects of *in vivo* ageing. Hayflick termed this failure to grow “senescence” a term which, for good or ill, has stuck.

Since Hayflick’s original discovery many different cell types have been shown to behave analogously to human fibroblast populations *in vitro*. Senescent cells have also been shown by a variety of methods to be present in many different tissues *in vivo*. This finally silenced the criticism, not uncommon among gerontologists until the 2000s, that senescence was simply a “tissue culture artefact” unworthy of study. Rather, senescence appears to be a ubiquitous characteristic of cells derived from regenerative somatic tissue and a primary causal mechanism of ageing and age-related disease.

However, as the list of cell types showing senescence has grown specialists in different branches of biology have found themselves grappling with questions once restricted to cytologists working predominantly on fibroblasts. How can senescent cells be simply and reproducibly detected both *in vitro* and *in vivo*? and to what extent can the same techniques be used in different cell types and different species? To address these questions this article will review the different markers available for the detection of senescent cells with an emphasis on the phenotypic aspects of senescence that originally validated them and how these in turn may inform on the senescent state in other contexts.

### Terminology

Clarity is essential when discussing the biology and growth dynamics of cells *in vitro* because the literature uses overlapping, sometimes contradictory, terms for the same cell populations (Hayflick, 1990). Without prejudice to other classification systems, this review will follow the usage of Shall (1987) in which cell cultures can be considered to fall into three types with respect to growth capacity: *Primary* cells are derived from normal tissue and display a limited lifespan in culture (although in some fields only the initial explant population is referred to as “primary”). “Primaries” are sometimes referred to as mortal cell cultures or as *cell strains-*the term originally used by Hayflick and favored by this author [although some researchers restrict use of it to subpopulations selected from a culture by cloning, e.g., Freshney (2005)]. *Continuous cell lines* are by definition composed of cells with an unlimited growth potential (mortal cell populations sometimes being termed *finite* cell lines). In the Shall typography *continuous lines* fall into two broad categories; *immortal cells* are non-tumorigenic, possess unlimited proliferative capacity and at that time were largely derived from rodents (e.g., 3T3 cells). By contrast, *transformed cells* represented the vast majority of cell lines produced from humans (e.g., HeLa or HepG2 cells). Transformed cell lines have the unlimited lifespan of continuous cell lines but form tumors in nude mice and show a number of other characteristics, such as growth in soft agar, which reflect this tumorigenicity *in vitro*. The underlying molecular mechanisms controlling growth and giving rise to this typography have become progressively clearer over the years (e.g., Shay et al., 1993; Zou et al., 2009).

Only cell strains spontaneously become senescent in culture, although senescence can be induced in cell lines through a variety of methods (typically, but not exclusively, involving DNA damage). Senescence is distinct from *quiescence*, a reversible growth arrested state which can be induced by the removal of serum from the medium of primary or immortal cells or through contact inhibition. In systems in which it is experimentally possible readily to separate the two states (e.g., epidermal keratinocytes grown in low calcium medium) senescence is distinct from terminal differentiation (Norsgaard et al., 1996).

“Senescence” is also regularly used in two distinct ways which can occasion further confusion. Senescence of the *entire culture*, originally called the “Phase III phenomenon” (Hayflick, 1979) is failure of the culture to proliferate under conditions which had previously allowed sustained cell growth. This growth is generally measured as Population Doublings (PDs) calculated as the number of times the cell population doubles in number during the course of culture Eq. 1.

$$PD=\frac{\log_{10}\left(\text{Numbers}\;\text{cells}\;\text{harvested}\right)-\log_{10}\left(\text{Number}\;\text{cells}\;\text{seeded}\right)}{\log_{10}2}$$ (1)

Primary cell populations typically go through 30–60 cumulative population doublings (CPD) by which time they are overwhelmingly composed of *senescent cells* (the second sense of the word as used by cytogerontologists). It is worth reiterating that a “senescent” culture is not necessarily free of growth competent cells, although they will be in a minority. This occurs because all that is required for the population to cease to expand and enter an apparently static phase is for the rate of production of newborn cells to be less than or equal to the population death rate (Kalashnik et al., 2000). The highly variable survival times of senescent populations from different cell types reported in the literature (days or weeks in the case of senescent HUVECs compared with months or years in the case of dermal fibroblasts) results in part from this dynamic.

The canonical feature of a senescent cell is its failure to divide in response to a conventional mitotic stimulus but this is also accompanied by radical alterations in cell physiology. Together these constitute the core phenotypes of cellular senescence from which the validity of all histological markers ultimately derive. Because it is possible to separate the altered phenotype from the cessation of growth in some contexts (e.g., the serial passage of adrenocortical cell strains) the failure of growth and the altered phenotype are considered separately below.

## The Core Phenotypes of Senescent Cells

### The Failure to Grow

Hayflick and Moorehead (1961) working with 25 independent strains of human fetal fibroblasts made the central finding that primary cultures cease to expand, and defined three distinct stages of growth *in vitro*. Phase I, the explant culture, was considered to terminate with the formation of the first confluent sheet of cells. Phase II was characterised by vigorous growth requiring repeated subculture. Phase III was a decline phase in which, after approximately 50 CPD, the cells showed cessation of mitosis, the accumulation of cell debris and degeneration of the culture (Hayflick, 1965). These three stages can be observed in a wide variety of different cell types but patience is required since 50 CPD takes roughly a year of continuous passage for fibroblasts (although fibroblast cultures with both greater and lesser proliferative capacities are common).

Hayflick’s original model carried two implicit assumptions. Firstly, that the decline in proliferative ability seen during “Phase III” resulted from cell death. Secondly, that the cultures studied were homogeneous populations of cells which were either all growing (in Phase I and II) or all non-growing (Phase III). Simple cell death as a cause of Phase III was quickly excluded by the demonstration that RNA synthesis (measured by incorporation of tritiated uridine) occurred in all population phases (Macieira-Coelho et al., 1966).

Although proliferative homogeneity was disproved a little later it is still widely assumed to be a feature of normal cell populations-perhaps because Hayflick’s original figure showing the three phase model is so regularly reproduced in tissue culture manuals. But Smith and Hayflick (1974) and Smith and Whitney (1980) demonstrated, by isolating and then culturing hundreds of individual WI38 and WI26 clones at different mass culture population doubling levels, that primary fibroblast cultures contain mixtures of clones with very variable growth potentials. Re-cloning a clone with a high divisional capacity produced sub-clones with a range of division potentials that gradually shifted towards smaller clones as the culture aged. Later studies examined the replicative capacity of thousands of isolated glial cells using the Pontén mini-cloning technique (Pontén et al., 1983). This assay is based on the use of custom tissue culture plates. These are created by the deposition of arrays of some hundreds of circular “islands” of palladium (typical areas 45,800 μm^2^ or 18,000 μm^2^), onto the central area of tissue culture plates that no longer allow cell adhesion (e.g., by being pretreated with agarose) whilst at the same time a ring of palladium is seeded around the outside (the overall effect is somewhat akin to islands of palladium in a lake or inland sea). In contrast to the clone isolation experiments of Smith and Hayflick (1974) or Smith and Whitney (1980) Pontén mini-cloning makes it possible to control for growth artefacts arising from low cell densities by simultaneously plating cells at normal densities on the ring of palladium forming the lake “shore” and cells at clonal densities on the islands (achieved by using a metal spacer to separate the low and high density subpopulations until they have attached). The fraction of non-dividing glial cells in these studies (n = 1760 clones) increases steadily from the earliest to the final passage (∼35 population doublings) in a very similar pattern to dermal fibroblasts and represented an early demonstration that senescence could be observed in cells from multiple different tissues.

Labelling studies complemented these single cell analyses. Cristofalo and Sharf (1973) carried out an analysis of the divisional kinetics of embryonic fibroblasts. This required 72 h 3H-thymidine pulse-labeling experiments at every passage throughout the culture lifespan. The fraction of cells which entered S phase was then estimated by autoradiography. They observed that unlabelled (senescent) cells were present in very young cultures, that a few labelled cells were present even in “senescent” cultures and that the fraction of unlabeled cells increased smoothly with serial passage. Additional experiments using WI38 fibroblasts demonstrated that although overall cell cycle length (T_c_) increased approximately two fold (from ∼19 to ∼30 h) with serial passage this was insufficient to explain the decline in the labelling index (Grove and Cristofalo, 1977). Thus the fraction of growing cells in the culture declines as the CPD level increases and fibroblast cultures are mixtures of label-excluding, senescent cells and their growing counterparts, the proportions of which alter as the cultures age. If these basics are neglected when studying a primary cell population then the results of any assay that generates an average value from the culture (in practice anything from Western blotting to next generation sequencing) become fundamentally insecure.

It is often unappreciated that such kinetic analyses underpin virtually all the more “sophisticated” techniques sometimes recommended for the identification of senescent cells today (González-Gualda et al. 2021). Although labelling studies have become progressively easier to perform through the replacement of 3H-thymidine first by 5-bromo-2′-deoxyuridine (allowing label incorporating cells to be detected by immunocytochemistry rather than autoradiography) and subsequently by 5-ethynyl-2′-deoxyuridine (which simplifies the technique still further through the use of a copper (I) catalyzed “click-chemistry” reaction between the 5-ethynyl group and an azide conjugated dye such as Alexa-594 or Pacific Blue) the basic principles and problems inherent to kinetic analyses are much the same today as they were in the 1970s.

The major problem associated with labelling is the need to ensure that the proportion of cells that take up the label accurately reflects the true culture growth fraction. A short labelling time (e.g., 30–90 min) underestimates this because serially passaged cultures are asynchronous and cells in G_1_, G_2,_ and M cannot incorporate label. Since the minimum S phase time is 6–8 h in cultures of MRC5 and WI38 fibroblasts regardless of CPD (Griffiths, 1984) roughly 70% of division competent WI38 cells will not be in S phase at any one time (T_c_ = 19–30 h). Long labels of the type used by Cristofalo and Sharf (1973) avoid this problem but overestimate the growth fraction. This is because growth fractions are calculated as the ratio of nuclei that have incorporated label over the total scored (typically 400 positive or 1,000 total nuclei giving the 95% confidence interval). With 72 h labels, cells in S phase when the label is introduced will go through G_2_ and M phase during the labelling period and will thus be scored as two separate positive nuclei; a process that could be repeated twice with typical T_c_ values.

A long label should thus be seen as providing a minimum value for the number of senescent cells present in a population (< 5% label incorporating nuclei on a 72 h label is often used as the definition of a fully senescent culture) whilst short labels are useful for determining the decline in a growth fraction during serial passage, particularly if comparing rates between multiple cell populations (Faragher et al. 1993). In theory, T_c_–T_s_ is the perfect labelling time but in practice almost never attempted whereas labelling times of 24 or 48 h are the hardest to interpret but occur in the literature with monotonous regularity (perhaps because they are simply easier to work into routine laboratory schedules). Minor problems with analogue labelling include the frustrating feature that media which contain high levels of thymidine (such as Ham’s F12 or some specialist media with “trade secret” compositions) can cause short labels to fail. BrdU labelling is also incompatible with the simple terminal transferase dUTP nick end labeling (TUNEL) apoptosis assay due to photolysis of any DNA that has incorporated BrdU. Ironically such labelled cells make excellent positive controls in a TUNEL assay.

Given that advantages and disadvantages are inherent in the use of thymidine analogues alternative methods based on the detection of proteins varying in amount and/or conformation through the cell cycle have also been employed to study senescence. Of these antibodies against Topoisomerase II and proliferating cell nuclear antigen (PCNA) have both been used in primary fibroblast cultures (Kill et al., 1994) but have similar problems to the use of thymidine analogues in accurately capturing the total culture growth fraction (PCNA detection is restricted to S phase and Topoisomerase II to G_2_/M). The most robust endogenous marker is probably Ki67, a multifunctional protein that is highly expressed in cycling cells but typically absent from cells in G_0_ (Gerdes et al., 1984). Although Ki-67 levels can vary in the first G_1_ following cell cycle re-entry after this the protein remains immunologically detectable in all cell cycle phases rendering it particularly useful in distinguishing senescent and growing cells within cultures. Using it Thomas et al. (1997) compared the rates of loss of the growth fraction in human dermal fibroblasts and peritoneal mesothelial cells which have very similar replicative lifespans *in vitro*. Mesothelial cells started out with a higher initial growth fraction than fibroblasts but lost it significantly faster (−2.2% Ki67 positive per PD compared with −0.89% for 2DD dermal fibroblasts). These findings illustrate the value of determining the kinetics of entry into senescence for the cell type studied; extrapolating from the fibroblast literature should be done with caution.

A feature common to all label exclusion methods is their inability to distinguish between quiescent and senescent cells. This is a major impediment *in vivo*, almost a fatal flaw, because the overwhelming majority of cells are quiescent most of the time. But, even here the technique can sometimes be used to advantage. Wolf and co-workers (Li et al. 1997) examined the effects of age and long-term caloric restriction on the accumulation of senescent cells in the murine lens (which has the advantage that cell proliferation is limited to a defined zone). Animals from 4 to 45 months of age were subject to one of two complementary procedures; either a two week infusion of 2 μg/g body weight per hour BrdU by osmotic minipump followed by histochemical detection of labelled cells in the proliferative zone and equator of the lens or simple sacrifice and isolation of lens epithelial cells which were then subjected to a colony size analysis of the type pioneered by Smith (Smith and Hayflick, 1974). Compared to their young counterparts old animals showed a highly significant (*p* < 0.001) increase in the number of label excluding cells present in the lens *in vivo*. These changes were mirrored *in vitro* where ∼40% of lens cells from 33 month old mice were senescent compared to ∼20% of cells from their 6 month old counterparts. The conclusion that the label excluding lens cells *in vivo* are the same population as the senescent lens cells *in vitro* seems inescapable and in both arms of the study calorie restriction significantly reduced their accumulation.

### Mechanisms of Senescence: Pathway Components as Specific Markers

In parallel with the study of the kinetics of senescence in primary populations researchers attempted to unravel the mechanistic basis of the process in individual cells. Early cell fusion experiments demonstrated that the phenotype of senescence was common between fibroblast strains from different donors, that it was dominant over growth in synkaryon fusions between growing and senescent cells (Littlefield, 1973) and that senescent nuclei or cytoplasm inhibited DNA synthesis in nuclei derived from young cells when partnered with senescent ones in heterokaryon experiments (Norwood et al. 1990). This work was complemented by the isolation of pairs of daughter cells generated by single mitotic events followed by determination of their proliferative potential. In a significant proportion of these pairs large proliferative differences between the daughters were observed indicating that human fibroblast senescence was controlled by the unequal partitioning of some controlling molecule which, although its nature at that time was unknown, was present at a concentration of less than 100 copies per cell (Jones et al., 1985). Taken together these results were consistent with a model in which senescence was controlled in the nucleus by a few elements and effected by one or more proteins which inhibited the transition of cells from G_1_-S phase.

As proposed by Olovnikov (1973), the finite elements regulating human fibroblast senescence eventually proved to be telomeres (which progressively shortened with cell division due to the absence of the repair enzyme telomerase). The primary effector protein, initially isolated by expression screening as senescent cell-derived inhibitor (sdi) was the now well-known cyclin dependent kinase inhibitor p21^waf^ (Noda et al. 1994). This core mechanistic model was validated by the simultaneous demonstrations that the reintroduction of telomerase into a range of human cell types prevented senescence (Bodnar et al. 1998; Wyllie et al. 2000) and that microinjection of blocking antibodies against p21^waf^ (Ma et al. 1999) or its major transcription factor p53 (Gire and Wynford-Thomas, 1998) rescued human fibroblasts from senescence. The observation that senescence in human fibroblasts is associated with activation of the DNA double-strand regulated ataxia-telangiectasia mutated (ATM) signalling pathway leading to the focal accumulation of the repair protein 53BP1 and phosphorylated histone H2AX (γ-H2AX) at telomeres (d'Adda di Fagagna et al. 2003; Gire et al. 2004) extended these studies and raised the possibility that the detection of components of this senescence mechanism could be used specifically to identify senescent cells *in vivo*.

This approach is attractive in principle. Techniques based on the measurement of telomere length distributions within a population have been used as evidence of cell turnover *in vivo* and, by inference, the presence of senescent cells in the populations sampled. Using this approach Allsopp et al (1995) demonstrated that human peripheral blood lymphocytes show an average reduction of 2 Kbp of telomere length over 50 years *in vivo* whereas brain tissue mean telomere length remains constant over the same period*.* But these methods are complicated by the fact that telomere shortening is not the sole, or even the predominant, mechanism by which mammalian cells enter senescence. Even in humans the telomere-dependent pathway is only used by a subset of cell types. Additional problems of interpretation have emerged as these techniques have grown in popularity.

Sedivy and co-workers (Herbig et al. 2006; Jeyapalan et al. 2007) using a baboon model were among the first to use this approach at the single cell level. Importantly, baboons had recently been shown to display age-associated telomere shortening in leukocytes that closely paralleled that seen in humans (Baerlocher et al. 2003) giving some confidence that telomere-dependent senescence was shared between the two species. An initial study of 30 baboons (age range 5–30 years, 15 males and five females) showed an age-related exponential increase in 53BP foci and γ-H2AX co-localisation with telomeres (so-called telomere dysfunction–induced foci or TIFs). 200–600 fibroblasts were scored from each biopsy with approximately a quarter of the cells positive for these markers, and thus presumably senescent, in the oldest animals. Less than 5% of cells were positive in the youngest members of the cohort.

However, a further study combining the analysis of baboon fibroblasts grown to senescence *in vitro* with the analysis of skin and muscle biopsies from animals of different ages revealed marked differences in the staining pattern of senescent cells *in vitro* and presumptively senescent cells *in vivo* (Jeyapalan et al. 2007). The panel of potential markers used included not only TIF detection but staining for ATM, HIRA, p53, and the cyclin dependent kinase inhibitors p21 and p16. The last was included because it was known to increase markedly in multiple strains of fibroblasts (WI38, IMR-90, MRC5, and NHDF) once senescence was established (Alcorta et al. 1996). HIRA staining was absent from muscle biopsies regardless of donor age but increased from ∼20% of fibroblasts in young skin biopsies to over 70% in those derived from 30-year-old baboons. However, the increase in HIRA staining occurred linearly, not exponentially, with age suggesting a distinct aetiology from that giving rise to TIFs. Whilst senescent baboon fibroblasts *in vitro* were uniformly p21 positive, with multiple γH2AX foci associated with ATM, 53BP1 and telomeres (over 80% of γH2AX foci colocalized with telomeric sequences) only 10% of these cells were p16 positive. But in skin biopsies fibroblasts stained for 53BP1 usually contained only a single focus (70% of 53BP1 positive cells in young animals and 65% in very old animals). *In vivo* p21 staining in dermal fibroblasts was largely absent but p16 staining was abundant. Such differences between senescent cells *in vitro* and *in vivo* should caution against simplistic “if it stains for *x* it’s a senescent cell” styles of thinking about any specific marker detection assay.

The most intellectually parsimonious hypothesis consistent with the phenotype of senescent baboon fibroblasts *in vivo* is that researchers are visualising a subset of cells that have been senescent for weeks or months. Stein et al. (1999) reported that whilst p21 progressively accumulates in IMR90 human fibroblasts serially passaged to senescence and is present exclusively in newly senescent cells it subsequently disappears. In contrast p16^Ink4a^ is initially absent but increases after IMR90s enter senescence and remains elevated for at least 2 months. Thus, p16^INK4a^ is an excellent potential pathway biomarker for senescent cells in situations where label exclusion or markers of increased cell size (q.v.) cannot readily be used. However some exceptions have been reported (Frescas et al. 2017a).

A particularly important study in this respect is that of Liu et al. (2009) who measured p16 levels in peripheral blood T lymphocytes from human donors (n = 170) aged from 18 to 80. p16^INK4a^ mRNA expression measured using Taqman quantitative RT-PCR showed a highly significant exponential relationship with donor chronological age (Log_2_[p16] gives *R* ^2^ = 0.4, *p* < 0.0001 *vs* donor age). Although a similar relationship was observed at the protein level multiple technical difficulties militated against using this measure. Perhaps predictably p16^INK4a^ levels increased more rapidly with age in smokers compared to non-smokers but provocatively the authors found a blunting of the age-p16 relationship with exercise intensity and duration. The positive association between levels of IL6 (a marker of frailty) and p16 could have indicated an element of “reverse causation” (frail people cannot exercise) however since it is now clear from rodent data that exercise facilitates the clearance of senescent cells by the immune system this can probably be discounted (Schafer et al. 2016).

Even though the measurement of telomere length has been significantly simplified in recent years by the introduction of quantitative-PCR (Montpetit et al. 2014) measurement of p16^INK4a^ message probably offers advantages over this technique for the detection of senescent cells because p16^INK4a^ appears regardless of whether the arrest mechanism is telomere dependent or independent and the message shows a greater dynamic range than mean telomere length (∼10 fold over 60 years compared to ∼2-fold for telomere length over the same period). Nonetheless any quantitative PCR assay remains a relatively costly system to invest in and optimize which begs the question-are there other aspects of the senescent cell phenotype that could be exploited to develop detection systems of equal value but greater ease of use particularly for small scale studies?

### The Altered Phenotype

#### Cytological Changes as Markers of Senescence

Senescent cultures of fibroblasts can be clearly distinguished from their growing counterparts by simple light microscopy because the cells are unmistakably bigger. Schneider and Mitsui (1976) found that the modal volume of cultured WI38 fibroblasts increased by approximately 40% from early passage to senescence (from 1930 ± 20 μm^3^ to 2,655 ± 234 μm^3^). Even larger increases occurred in both RNA (110%) and protein content (∼80%). Sherwood et al. (1988) studied IMR90 human fibroblasts using multiparameter flow cytometry and found that mean cell size shifted approximately two-fold between passages 28 and 53. Consistent with earlier work (Cristofalo and Sharf, 1973; Smith and Whitney, 1980) a few such large cells were present in early passage cultures. However senescent cultures were dominated by these large cells whilst their cycling counterparts, even in late-passage cultures, remained relatively small. Similar patterns emerge with other adherent cell types undergoing senescence (e.g., mesenchymal stem cells see Adewoye et al. 2020). Thus, in principle it is possible to identify some types of senescent cells simply by this increase in size coupled with non-invasive markers such as autofluorescence (Poot et al. 1985; Bertolo et al. 2019). But not all cell types show this alteration in cell size at senescence, T cells being the best-known exception (Perillo et al., 1989). Thus, size alone cannot be used as a marker of senescence unless there is prior evidence that hypertrophy accompanies growth arrest.

In senescent fibroblasts this increase in size and protein content is accompanied by a range of ultrastructural changes particularly increased nuclear size, nuclear abnormalities, larger autophagic vacuoles and increased numbers of lysosomes (Lipetz and Cristofalo, 1972; for review see; Stanulis-Praeger, 1987). This latter change is of particular importance for the detection of senescence. Whilst Cristofalo and Kabakjian (1975) were probably the first researchers to report increased activities for the lysosomal enzymes acid phosphatase and β glucuronidase as WI38 cells entered senescence. Campisi and co-workers (Dimri et al., 1995) working with seven different strains of human fibroblasts (including WI-38) modified a histochemical assay for lysosomal β-galactosidase activity to develop probably the most widely used technique for the detection of senescent cells. This colorimetric assay is based on cleavage of the soluble, colourless lactose analogue X-gal (5-bromo-4-chloro-3-indolyl-β-D-galactopyranoside) to form an insoluble blue precipitate (5,5′-dibromo-4,4′-dichloro-indigo). By shifting the pH of the reaction buffer away from the β-galactosidase optimum of pH4 to pH6 the researchers rendered the enzyme less efficient and ensured that only cells with lots of lysosomal β-galactosidase could cleave enough X-gal to generate visible precipitates. Since this effectively limits detectable precipitate to large cells (more formally cells with a high lysosomal mass) which are usually senescent, it is perhaps unsurprising that Dimri et al. (1995) reported a high correlation between cells that were “senescence-associated β-galactosidase” (SAβ-gal) positive and cells that were label excluding on 3H-thymidine long labels.

The original SAβ-gal staining technique is simple and robust, rendering it suitable for use in a variety of settings. Given the range of lysosomal enzymes and substrates available for them this approach is also applicable to a wide range of other enzymes. We found some years ago (Dropcova and Faragher, unpublished observations) that when the optimum pH for the enzyme is shifted both dipeptidyl peptidase-4 (CD26) and amino peptidase M show similar “senescence associated” staining patterns to β-galactosidase (unfortunately the coupling chemistry produces a “red on yellow” staining pattern that is harder to score than SAβ-gal). Recently, Hildebrand et al. (2013) showed that the lysosomal enzyme α-fucosidase, is upregulated in cells made senescent through a range of different techniques. Enzyme activity can be visualised using 5-bromo-4-chloro-3-indolyl-α-L-fucopyranoside (X-fuc) in an incubation buffer similar to that for SAβ-gal (but shifted to pH 5.0 not pH6.0). This gives a blue stain similar to X-gal but with the advantage that α-fucosidase induction is somewhat stronger at senescence, particularly in murine cells rendered senescent by drug treatment.

Although the original SAβ-gal histochemical techniques use a paraformaldehyde fixation step a range of fluorescent substrates are also available for β-galactosidase (Kurz et al. 2000; Okamoto et al. 2006; Filho et al. 2018) allowing the visualisation of SAβ-gal in live cells (provided the lysosomes are temporarily alkalinised for example by treatment with bafilomycin A_1_). One substrate (5-dodecanoylaminofluorescein di-β-D-galactopyranoside) has been successfully used for flow cytometry (Kurz et al. 2000; Noppe et al. 2009) opening up the possibility of flow sorting viable senescent cells. Solution phase variations on these techniques are also available for another style of quantitative read out (Cho and Hwang, 2011).

Users of these techniques should be aware of two potential complications. Firstly, there can be significant delay between entry into senescence and detectable SAβ-gal staining. This was first reported by Thomas et al. (1997) in AGO7086A human mesothelial cells dual stained for Ki67 and SAβ-gal although it was not observed in HUVECs or RPE cells analyzed using identical techniques (Rawes et al., 1997; Kalashnik et al. 2000). Secondly, an increase in the amount of β-galactosidase at senescence is necessary for visualisation. Kurz et al. (2000) using HUVEC showed a 15-fold increase in the amount of enzyme from early passage to senescence; an increase that was paralleled by the increase in lysosomal mass. This does not require an increase in cell size explaining why it is possible, though rarely attempted, to identify senescent T cells by SAβ-gal staining (Gerland et al. 2004; Yang et al. 2018) even though their nucleocytoplasmic ratio is unchanged. However, senescent cells with very short post-mitotic survival times or unaltered lysosome contents will be effectively invisible.

Whilst Stein et al. (1999) had noted that the increase in p16^INK4^ roughly parallels the increase in cell size and SAβ-gal in senescing human fibroblasts a more comprehensive temporal analysis was undertaken by Cho and Hwang (2011). Using MCF-7 breast cancer cells rendered senescent by exposure to adriamycin the researchers measured cell cycle-related proteins (including p21 and p53 levels), SAβ-gal activity, cell volume and autofluorescence among other markers. Consistent with earlier studies the levels of p21 and p53 increased within the first 24 h following exposure to the drug whilst cell size increased only for the first 2 days. In contrast, autofluorescence increased 5-fold above baseline levels over 8 days in a roughly linear fashion whilst the number of SAβ-gal positive cells increased in a sigmoidal fashion before stabilising after 6 days. Nonetheless, SAβ-gal activity continued to increase beyond this from an eightfold increase over baseline at day 6 post treatment to a maximum 14-fold increase by day 8. Differences of this scale are in principle detectable simply by immunocytochemical staining for the enzyme itself although this has rarely been attempted (Joselow et al. 2017).

Thus, although senescent cells from any tissue are potentially detectable by variants of catalytic histochemistry for lysosomal enzyme activity, timing is central to the absolute value of the readout (raising issues of standardisation) and to be visualised cells must 1) have a substantial increase in lysosomal mass irrespective of hypertrophy and 2) have spent sufficient time senescent for enzyme activity to have built up to detectable levels. It should also be borne in mind that any other alterations in cell physiology that meet these criteria will generate a false positive signal. Most famously this can occur in primary fibroblast cultures held confluent or immortal cultures at high cell densities (Severino et al. 2000) and illustrates an important quirk of the routine employment of the technique.

As it stands, the research literature is heavily slanted towards reports of the detection of SAβ-gal *in vitro* rather than *in vivo*. This is ironic because the key advantage of the technique is that it gives researchers the capacity to detect senescent cells in tissue samples. When first introduced SAβ-gal was effectively the only assay which allowed senescent cells to be distinguished from their quiescent counterparts in this context. Ironically, in some publications (e.g., Xia et al. 2020) SAβ-gal has been used *in vitro* to check if a cell type of interest is senescent whilst its senescence *in vivo* is established using another method (e.g., p16^INK4a^ staining). It is questionable whether using SAβ-gal staining like this is adding much value to the study.

Unfortunately, reviews of SAβ-gal staining also tend to gloss over the key question of how such stained sections should be scored. This is regrettable, particularly for those using it for the first time because no real consensus has yet emerged around scoring (which raises issues of inter study comparability). A few examples illustrate the range of approaches that have been adopted.

Originally, Dimri et al. (1995) relied on blind scoring of 20 dermal sections from human donors of varying age by a specialist pathologist with the staining frequency presented as a simple scale from minus (indicating no staining) through to “+ + +” (positive cells in all sections of dermis, multiple clusters in all sections of epidermis). Similarly, Paradis et al. (2001) used independent assessment by two pathologists to gauge the localization and number of positive cells in 57 biopsies from normal and abnormal human livers. SAβ-gal staining was classified simply as either “absent” (no or < 10% SAβ-gal positive cells visible) or “present” (> 10% SAβ-gal positive cells visible). Simultaneous review of sections was adopted if the pathologists differed in their opinion.

Kim et al. (2008) took a much more quantitative approach to scoring SAβ-gal in nucleus pulposus chondrocytes within intervertebral disc sections from 25 patients. Here every chondrocyte on the whole section was counted (under × 200 magnification with Nuclear Fast Red counter-stain) and the SAβ-gal positive fraction presented as a percentage. A similarly rigorous histomorphometric approach was used by Gruber et al. (2007) in 57 human disc samples giving an overall incidence of 29.9% (SD ± 24.8, range from 0 to 92%.) positive cells.

Berkenkamp et al. (2014) studied the frequency of senescence in mouse renal epithelial cells *in vivo* using a semi-quantitative approach in which the frequency of SAβ-gal positive cells in 10 random fields of view within representative kidney sections were scored. This was sufficient to yield a statistically significant difference (*p* < 0.05) between young (3–5 months) or old (18 + month) animals.

In contrast Melk et al. (2003) studying cell senescence in Fischer 344 rat kidneys *in vivo* quantified SAβ-gal staining simply by imaging kidney sections using Image-Pro Plus Software. A set of slides without the eosin counterstain the group normally employed were photographed, average staining density for the whole section was calculated and the mean staining density of two independent experiments, in arbitrary units, was used as the basis for further calculations. Even this simple approach proved adequate for the demonstration of a significant difference in SAβ-gal staining levels between young (9 months) and old (24 months) rats (0.008 ± 0.003 *vs* 0.020 ± 0.007 arbitrary units respectively, *p* < 0.005).

#### Metabolic Changes as Markers of Senescence

Alterations in the amounts and activity of lysosomal enzymes are a subset of the changes that occur in cellular metabolism with senescence. Increased lysosomal size results, at least in part, from dysregulated proteostasis, a key hallmark of ageing. Misfolded proteins are degraded less effectively and form aggregates which, when internalised by lysosomes, contribute to the highly cross-linked and complex materials known collectively as lipofuscin or ceroid (Yin, 1996; Moreno-García et al. 2018). In classical histology these terms are distinct referring to material accumulating within post mitotic or mitotic cell types respectively but colloquially the “ceroid” within senescent cells is often simply called “lipofuscin”. This highly heterogenous “junk” material is responsible for the increased autofluorescence of senescent cells and its presence gives another visualisation option. In essence, rather that staining for the elevated activity of lysosomal breakdown enzymes, simply stain for the “junk” they are trying to break down instead.

This approach can be particularly useful if trying to visualize senescent cells in tissue samples fixed in ways that inactivate lysosomal enzymes (e.g., formalin-fixed paraffin-embedded blocks). As noted earlier immediately following the onset of senescence different markers build up to detectable levels at different rates (Cho et al., 2011) but since the majority of senescent cells *in vivo* will not usually be immediate entrants this is probably not a serious flaw under normal circumstances. Melk et al. (2003) correlated lipofuscin levels (graded independently by two observers) with SAβ-gal staining in the tubular epithelium of their rat samples and found a highly significant association (*p* < 0.001) in both young and old animals.

Melk et al. (2003) used a classic histological stain (based on the periodic acid-Schiff reaction) to visualie lipofuscin. Recently Georgakopoulou et al. (2013) used another (Sudan Black B counterstained with Nuclear Fast Red) to co-localise lipofuscin and SA-β-gal in senescent cells. Building on the success of this, but conscious of the difficulties of interpretation of traditional lipofuscin staining, the group undertook the *de novo* synthesis of a series of Sudan Black B analogues coupled to biotin which they validated in a range of test systems against both proliferation markers (Ki67, BrdU) and SAβ-gal (Evangelou et al. 2017). This approach allowed the ready visualization of lipofuscin using a peroxidase conjugated anti-biotin antibody and diaminobenzidine (DAB) visualization (although any of a range of anti-biotin detection systems, such as those based on avidin would also probably have worked). In γ-irradiated human fibroblasts *in vitro* one such analogue (GL13) was used to visualize the kinetics of lipofuscin accumulation giving a timescale broadly similar to that observed by Cho et al. (2011) for autofluorescence. However, all enzyme-based visualisation systems produce staining of variable intensity, so the absolute numbers of senescent cells visualized by this type of lipofuscin detection may differ significantly between researchers depending on simple variables such as the incubation time.

Similarly, Masaldan et al. (2018) building on prior work demonstrating the accumulation of iron within senescing IMR90 fibroblasts and HUVECs (Killilea et al. 2003) studied iron accumulation in mouse embryonic fibroblasts rendered senescent by sublethal γ-irradiation, serial passage or oncogene activation. After 10 days of growth arrest all these types of senescent rodent cells accumulated extremely large amounts of intracellular iron (∼15–20 fold compared to growth competent controls). Human fibroblasts and prostate epithelial cells rendered senescent by irradiation or serial passage also accumulated lesser quantities of iron after longer periods of growth arrest (∼3.3 to 8.4-fold 21 days post senescence depending upon the cell type and mechanism by which senescence was induced). This accumulation of iron in senescent rodent cells results from the upregulation of the transferrin receptor and a tenfold elevation of ferritin. This in turn was shown to result from impaired lysosome-mediated degradation of this key iron storage protein. Practically, this significant difference in iron handling between the senescent and growing states allowed the group to visualize senescent cells in mouse liver by staining with a rabbit polyclonal antibody against ferritin (in combination with an HRP labelled secondary antibody, DAB visualization and a haematoxylin counterstain). Serial liver sections from young and old animals were stained for SA-β-gal and ferritin respectively and scored by observing four independent fields of view. These findings open up a wide range of potential staining strategies for senescent cells since techniques for the visualization of iron in histological samples are well developed and encompass everything from classical stains such as Pearl’s Prussian blue through to quantum dots and can be used in formalin-fixed paraffin-embedded material (Meguro et al., 2007; Duan et al.,2018; van Duijn et al. 2013).

Advanced Glycation End products (AGEs) are often grouped with lipofuscin but unlike the latter can form both extra and intracellularly. They are a heterogeneous group of compounds typically formed by the nonenzymatic (Maillard) reactions of glucose with proteins or lipids. To date limited work has been done to address whether the visualization of AGEs can be used to detect senescent cells but initial studies by Sell et al. (1998) suggest that this should be possible. The authors looked at the accumulation of the AGE pentosidine in strains of reticular and papillary fibroblasts derived from a single donor and passaged to senescence as well as peripheral blood T lymphocytes from 27 donors of varying ages (17–97 years) and states of health. Levels of pentosidine were quantified by HPLC and increased approximately three-fold in both types of fibroblast over the culture replicative lifespan (*p* < 0.0007) whilst T lymphocytes showed a highly significant increase in pentosidine level with donor age (*p* < 0.0003). In related work, Kueper et al (2007) demonstrated that the intermediate filament protein vimentin aggregates in human dermal fibroblasts *in vitro* due to its modification by pentosidine and carboxymethyllysine (as well as other AGES such as pyrraline and carboxyethyllysine). More recently Frescas et al. (2017b) conducted a deliberate screen for senescence-specific markers by immunising mice with mouse lung fibroblasts rendered senescent using bleomycin. Human fibroblasts rendered senescent by IR were used as a secondary screen alongside untreated controls. One of the IgMs generated by this approach (9H4) showed approximately a two-fold greater level of staining on senescent cells in this screen. Subsequent Western blotting showed that 9H4 recognises a modified form of vimentin that is presented at the cell surface and then secreted (possibly facilitating the clearance of senescent cells by the innate immune system). The secreted vimentin was modified by a malondialdehyde adduct on cysteine 328. This is provocative because malondialdehyde is an end product of lipid peroxidation that subsequently gives rise to immunologically detectable AGEs (Sajithlal and Gowri Chandrakasan, 1999). Since antibodies to pentosidine and other AGEs such as carboxymethyl lysine are commercially available it would seem likely that senescent cells could be identified histochemically using this type of approach.

Vimentin is not the first example of a modified form of a normal protein found at the surface of senescent cells. Porter et al. (1990), Porter et al. (1992) had previously reported the generation of three monoclonal antibodies (SEN-1, SEN-2, and SEN-3) which recognized epitopes on fibronectin only exposed when human fibroblasts become senescent. These antibodies (tragically lost in a laboratory accident shortly thereafter Jim Smith-personal communication) could detect senescent human fibroblasts, keratinocytes and mammary epithelial cells *in vitro* and *in vivo* in a species-specific manner. If the secretion of modified proteins is a means of signalling for immune clearance then it is likely that there will be significant variations between cell types, species and perhaps individuals as is seen for other components of the Senescence Associated Secretory Phenotype (SASP).

Changes in cellular components as a consequence of dysregulated proteostasis should be conceptually distinguished from those which arise from the transcriptional or post transcriptional regearing that accompanies entry into senescence. These latter changes offer a great many potential markers for the senescent state but can vary widely in the same cell type depending on the inducing stimulus. Nelson et al. (2014) used a microarray-based approach (based on the Affymetric Human Genome U133 Plus 2.0 GeneChip) to compare the transcriptomes of IMR90 fibroblasts rendered senescent either by serial passage or by retroviral infection with H-RAS-V12 (oncogene induced senescence). Compared to proliferation competent controls 5,424 genes were differentially expressed at replicative senescence and 3,188 in OIS. However, there was only moderate overlap between the two states (∼33% of those genes altered by serial passage were altered by OIS but ∼56% of these was also altered by classical replicative senescence). Interestingly although both types of senescence were associated with p16^INK4a^ upregulation the cluster of genes associated with this pathway showed significant differences between the two states (69 out of 118 transcripts commonly downregulated and only 6 out of 31 transcripts commonly upregulated). This has important implications for the use of specific pathway components, as opposed to end points, as pan-specific markers of senescence between different states and tissues.

## Future Challenges in Senescent Cell Detection

It has been clear for decades that senescence occurs in fibroblast cultures from many mammalian species (Röhme, 1981) but the cell senescence literature remains heavily slanted towards humans and rodents. This is unfortunate because there are several other species which serve as excellent models for human ageing changes or age-related diseases. Given the evidence for the key role played by senescence in ageing the routine detection of senescent cells in these models will become increasingly important but the relevant literature base is currently weak or non-existent.

This is a potential problem because comparative studies of rodent and human fibroblasts show that extrapolation of senescence markers other than label exclusion from one species into another should be done with caution unless there are prior data available. The three models below have been selected to illustrate both the potential gains and the current issues involved in broadening the detection of senescent cells beyond humans and mice.

In the pig, which provides a good model for cardiovascular and intravertebral disc ageing in humans as well as a new model for human progeria (Dorado et al. 2019) there is a small but coherent evidence base for senescent cell detection. Senescent cells can be visualized by the SAβ-gal assay post formaldehyde-fixation (Shi et al. 2018) and serially passaged foetal porcine fibroblasts enter telomere-dependent senescence in a manner that closely resembles that seen in humans. This means that telomere shortening, TIF detection, p16^INK4a^ and p21^WAF^ can all potentially be used as markers for the senescent state in porcine tissue *in vivo* (Fukuda et al. 2012; Ji et al. 2012; Donai et al. 2014; Shi et al. 2018). However, Oh et al. (2007) demonstrated that it is possible to produce immortal clones of fibroblasts from both mini-pigs and three-way crossbred meat animals simply by culturing the cells using the 3T3 passage technique. This is not possible using human fibroblasts (McCormick and Maher, 1988) and suggests either that the finding is artefactual or that the molecular pathways regulating entry into senescence are not identical in the two species.

The ageing horse is a particularly valuable model for the types of age-related tendon injuries seen in older humans due to the many similarities between the human Achilles and the equine superficial digital flexor tendon in structure, matrix composition and function. Cultures of human tenocytes from aged and functionally degenerate Achilles show upregulation of p16^INK4a^ protein and a five-fold increase in the SAβGal positive fraction compared to healthy controls (Kohler et al. 2013) a finding that renders the detection of senescent cells in the equine tendon an important goal but studies on senescent cells in equines are limited. Foetal horse kidney cells can be immortalised with SV40 large T antigen (Maeda et al. 2007) indicating p53 and pRb dependent checkpoints and adult equine fibroblasts can be induced to proliferate continuously by the stabilisation of telomere length (Vidale et al. 2012) suggesting equines show telomere dependent senescence. SA-β-gal, p16^INK4a^ protein and γH2AX foci are all detectable in equine chondrocytes following γ irradiation (Copp et al. 2021) suggesting that these would also be useful markers. But as with most other animal models the kinetics of accumulation of these markers post entry into senescence remain unstudied. The type of studies carried out by Cho et al. (2011) are sorely needed.

Selective breeding has created hundreds of dog breeds which vary widely in lifespan presenting an exceptional opportunity to identify pathways associated with ageing. Unfortunately, whilst there is evidence for most of the known hallmarks of ageing in canines (Sándor and Kubinyi, 2019) research on the presence and phenotype of senescent cells in dogs is fragmentary. Whilst breed-specific telomere length has been shown to be a strong predictor of average life span and pathology (Fick et al. 2012) and SA-β-gal has been used to identify senescent canine fibroblasts *in vitro* there are clearly important differences between human and canine senescence. As with pigs, You et al. (2004) were able to isolate spontaneously immortalized clones from cultures of dog embryonic fibroblasts *in vitro*. These were shown to have mutations in either p53 or p16^INK4a^ suggesting that these pathways control senescence in canine fibroblasts but appear to be “leaky” compared to humans. A single study suggests that the proinflammatory phenotype of senescent canine dermal fibroblasts may also differ significantly from those of humans and rodents (Jimenez et al. 2020). Given the potential value of the model for biogerontology systematic characterisation of canine senescence at the cellular level would clearly be extremely valuable.

Two issues are likely to arise as the detection of senescent cells in different species becomes more commonplace. Firstly, there is a distinct possibility that the relative importance of senescence as an ageing mechanism may differ markedly between them as a result of evolutionary history. Ageing exists as a result of the declining force of natural selection with age and results from two non-exclusive modes of evolutionary gene action. Senescence is thought to have arisen through one of these, antagonistic pleiotropy. This is the selection for alleles or processes that enhance the reproductive success of organisms early in life but which have deleterious effects in the later life course. But, ageing also occurs as a consequence of the inability of natural selection to remove late acting deleterious alleles; a mode of gene action known as mutation accumulation. A wealth of experimental data shows that the relative contributions of antagonistic pleiotropy and mutation accumulation to the evolution of ageing vary widely between species.

One reason for this variation may be that selectively neutral deleterious alleles show frequencies that are influenced by genetic drift and the rate of drift is heavily influenced in turn by effective population size. Unlike most species, humans have undergone both significant genetic bottlenecks and have shifted their survivorship curve from a Type-II population to a Type-I population. This significantly increases drift and thus may well alter the relative importance of cellular senescence as an ageing mechanism between species (Overall and Faragher 2019). Put crudely, humans are evolutionary outliers.

Secondly, different species have very different lifespans which foregrounds the relative extent to which markers of chronological and biological ageing are uncoupled between them. Evidence is emerging from the study of human senescence that some markers better reflect absolute chronological time than cell replication frequency. Maier and colleagues (Waaijer et al. 2012) measured the fraction of senescent cells (*via* p16^INK4a^ immunostaining) in the dermis and epidermis of a selected sub-group of subjects from the Leiden Longevity Study who were biologically younger than age-and environmentally matched controls (n = 89 per group). The levels of senescent cells correlated closely with the pathological status of the donors whilst age and environment matched controls showed higher levels of senescent cells than the biologically young group. The authors concluded that p16 ^INK4a^ is a marker of biological time. Further studies using the same experimental design (Waaijer et al. 2016) investigated the relationship between markers of DNA damage (micronuclei, p53BP1 damage foci and telomere associated foci) and health status (n = 40 in each group). There p53BP1 and telomere associated damage foci, but not micronuclei, increased significantly with chronological age but, unlike p16 ^INK4a^ there was no association between these markers of DNA damage and the health status of the subjects examined. The authors concluded that p53BP1 and telomere associated damage foci effectively measured human chronological age. Studies of this type are likely to be necessary in other species to properly assess the contribution that cell senescence makes to their overall ageing. However, forewarned is at least forearmed and the range of simple detection techniques available suggests that the gaps in our current understanding can be closed much faster in novel systems than was the case when senescence markers were first systematically applied to human systems in the 1970s and 1980s.

## References

[B1] Adewoye A. B. Tampakis D. Follenzi A. Stolzing A. (2020). Multiparameter Flow Cytometric Detection and Quantification of Senescent Cells *In Vitro* . Biogerontology 21, 773–786. 10.1007/s10522-020-09893-9 32776262 PMC7541365

[B2] Alcorta D. A. Xiong Y. Phelps D. Hannon G. Beach D. Barrett J. C. (1996). Involvement of the Cyclin-dependent Kinase Inhibitor P16 (INK4a) in Replicative Senescence of normal Human Fibroblasts. Proc. Natl. Acad. Sci. 93, 13742–13747. 10.1073/pnas.93.24.13742 8943005 PMC19411

[B3] Allsopp R. C. Chang E. Kashefi-Aazam M. Rogaev E. I. Piatyszek M. A. Shay J. W. (1995). Telomere Shortening Is Associated with Cell Division *In Vitro* and *In Vivo* . Exp. Cell Res. 220, 194–200. 10.1006/excr.1995.1306 7664836

[B4] Baerlocher G. M. Mak J. Röth A. Rice K. S. Lansdorp P. M. (2003). Telomere Shortening in Leukocyte Subpopulations from Baboons. J. Leukoc. Biol. 73, 289–296. 10.1189/jlb.0702361 12554806

[B5] Berkenkamp B. Susnik N. Baisantry A. Kuznetsova I. Jacobi C. Sörensen-Zender I. (2014). *In Vivo* and *In Vitro* Analysis of Age-Associated Changes and Somatic Cellular Senescence in Renal Epithelial Cells. PLoS One 9, e88071. 10.1371/journal.pone.0088071 24505380 PMC3913727

[B6] Bertolo A. Baur M. Guerrero J. Pötzel T. Stoyanov J. (2019). Autofluorescence Is a Reliable *In Vitro* Marker of Cellular Senescence in Human Mesenchymal Stromal Cells. Sci. Rep. 9, 2074. 10.1038/s41598-019-38546-2 30765770 PMC6376004

[B7] Bodnar A. G. Ouellette M. Frolkis M. Holt S. E. Chiu C. P. Morin G. B. (1998). Extension of Life-Span by Introduction of Telomerase into normal Human Cells. Science 279, 349–352. 10.1126/science.279.5349.349 9454332

[B8] Carrel A. (1913). Artificial Activation of the Growth *In Vitro* of Connective Tissue. J. Exp. Med. 16, 14–19. 10.1084/jem.17.1.14. 10.1084/jem.17.1.14 PMC2125019 19867620

[B9] Cho S. Hwang E. S. (2011). Fluorescence-based Detection and Quantification of Features of Cellular Senescence. Methods Cell Biol. 103, 149–188. 10.1016/b978-0-12-385493-3.00007-3 21722803

[B10] Cho S. Park J. Hwang E. S. (2011). Kinetics of the Cell Biological Changes Occurring in the Progression of DNA Damage-Induced Senescence. Mol. Cell 31, 539–546. 10.1007/s10059-011-1032-4 PMC3887620 21533552

[B11] Copp M. E. Flanders M. C. Gagliardi R. Gilbertie J. M. Sessions G. A. Chubinskaya S. (2021). The Combination of Mitogenic Stimulation and DNA Damage Induces Chondrocyte Senescence. Osteoarthritis Cartilage 29, 402–412. 10.1016/j.joca.2020.11.004 33227437 PMC7925350

[B12] Cristofalo V. J. Sharf B. B. (1973). Cellular Senescence and DNA Synthesis. Exp. Cel. Res. 76, 419–427. 10.1016/0014-4827(73)90394-7 4568162

[B13] Cristofalo V. Kabakjian J. (1975). Lysosomal Enzymes and Aging *In Vitro*: Subcellular Enzyme Distribution and Effect of Hydrocortisone on Cell Life-Span☆. Mech. Ageing Develop. 4, 19–28. 10.1016/0047-6374(75)90004-4 1142849

[B14] Dimri G. P. Lee X. Basile G. Acosta M. Scott G. Roskelley C. (1995). A Biomarker that Identifies Senescent Human Cells in Culture and in Aging Skin *In Vivo* . Proc. Natl. Acad. Sci. 92, 9363–9367. 10.1073/pnas.92.20.9363 7568133 PMC40985

[B15] Donai K. Kiyono T. Eitsuka T. Guo Y. Kuroda K. Sone H. (2014). Bovine and Porcine Fibroblasts Can Be Immortalized with Intact Karyotype by the Expression of Mutant Cyclin Dependent Kinase 4, Cyclin D, and Telomerase. J. Biotechnol. 176, 50–57. 10.1016/j.jbiotec.2014.02.017 24589663

[B16] Dorado B. Pløen G. G. Barettino A. Macías A. Gonzalo P. Andrés-Manzano M. J. (2019). Generation and Characterization of a Novel Knock-In Minipig Model of Hutchinson-Gilford Progeria Syndrome. Cell Discov. 5, 16. 10.1038/s41421-019-0084-z 30911407 PMC6423020

[B109] Duan L. Wang P. Ren M. Liao F. (2018). Selective Probes for Ferric Ion: A Highly Fluorescent Nitrogen-Doped Carbon Quantum Dots. Inorg. Chem. Commun. 96, 111–115. 10.1016/j.inoche.2018.07.045

[B17] Ebeling A. H. (1942). Dr. Carrel's Immortal Chicken Heart. Sci. Am. 166, 22–24. 10.1038/scientificamerican0142-22

[B18] Evangelou K. Lougiakis N. Rizou S. V. Kotsinas A. Kletsas D. Muñoz-Espín D. (2017). Robust, Universal Biomarker Assay to Detect Senescent Cells in Biological Specimens. Aging Cell 16, 192–197. 10.1111/acel.12545 28165661 PMC5242262

[B19] Fagagna F. d. A. d. Reaper P. M. Clay-Farrace L. Fiegler H. Carr P. Von Zglinicki T. (2003). A DNA Damage Checkpoint Response in Telomere-Initiated Senescence. Nature 426, 194–198. 10.1038/nature02118 14608368

[B20] Faragher R. G. Kill I. R. Hunter J. A. Pope F. M. Tannock C. Shall S. (1993). The Gene Responsible for Werner Syndrome May Be a Cell Division "counting" Gene. Proc. Natl. Acad. Sci. 90, 12030–12034. 10.1073/pnas.90.24.12030 8265666 PMC48119

[B21] Fick L. J. Fick G. H. Li Z. Cao E. Bao B. Heffelfinger D. (2012). Telomere Length Correlates with Life Span of Dog Breeds. Cell Rep. 2, 1530–1536. 10.1016/j.celrep.2012.11.021 23260664

[B22] Frescas D. Hall B. M. Strom E. Virtuoso L. P. Gupta M. Gleiberman A. S. (2017a). Murine Mesenchymal Cells that Express Elevated Levels of the CDK Inhibitor p16(Ink4a) *In Vivo* Are Not Necessarily Senescent. Cell Cycle 16, 1526–1533. 10.1080/15384101.2017.1339850 28650766 PMC5584871

[B23] Frescas D. Roux C. M. Aygun-Sunar S. Gleiberman A. S. Krasnov P. Kurnasov O. V. (2017b). Senescent Cells Expose and Secrete an Oxidized Form of Membrane-Bound Vimentin as Revealed by a Natural Polyreactive Antibody. Proc. Natl. Acad. Sci. USA 114, E1668–E1677. 10.1073/pnas.1614661114 28193858 PMC5338544

[B24] Freshney R. I. (2005). Culture of Animal Cells: A Manual of Basic Technique. 5th Edition. Hoboken, NJ: Wiley–Blackwell. 10.1002/0471747599.cac019

[B25] Fukuda T. Katayama M. Yoshizawa T. Eitsuka T. Mizukami H. Nakagawa K. (2012). Efficient Establishment of Pig Embryonic Fibroblast Cell Lines with Conditional Expression of the Simian Vacuolating Virus 40 Large T Fragment. Biosci. Biotechnol. Biochem. 76, 1372–1377. 10.1271/bbb.120155 22785463

[B26] Georgakopoulou E. A. Tsimaratou K. Evangelou K. Fernandez Marcos P. J. Zoumpourlis V. Trougakos I. P. (2013). Specific Lipofuscin Staining as a Novel Biomarker to Detect Replicative and Stress-Induced Senescence. A Method Applicable in Cryo-Preserved and Archival Tissues. Aging (Albany NY) 5, 37–50. 10.18632/aging.100527 23449538 PMC3616230

[B27] Gerdes J. Lemke H. Baisch H. Wacker H. H. Schwab U. Stein H. (1984). Cell Cycle Analysis of a Cell Proliferation-Associated Human Nuclear Antigen Defined by the Monoclonal Antibody Ki-67. J. Immunol. 133, 1710–1715. 6206131

[B28] Gerland L.-M. Genestier L. Peyrol S. Michallet M.-C. Hayette S. Urbanowicz I. (2004). Autolysosomes Accumulate during *In Vitro* CD8+ T-Lymphocyte Aging and May Participate in Induced Death Sensitization of Senescent Cells. Exp. Gerontol. 39, 789–800. 10.1016/j.exger.2004.01.013 15130673

[B29] Gire V. Roux P. Wynford-Thomas D. Brondello J.-M. Dulic V. (2004). DNA Damage Checkpoint Kinase Chk2 Triggers Replicative Senescence. EMBO J. 23, 2554–2563. 10.1038/sj.emboj.7600259 15192702 PMC449769

[B30] Gire V. Wynford-Thomas D. (1998). Reinitiation of DNA Synthesis and Cell Division in Senescent Human Fibroblasts by Microinjection of Anti-p53 Antibodies. Mol. Cell. Biol. 18, 1611–1621. 10.1128/mcb.18.3.1611 9488478 PMC108876

[B31] González-Gualda E. Baker A. G. Fruk L. Muñoz-Espín D. (2021). A Guide to Assessing Cellular Senescence *In Vitro* and *In Vivo* . FEBS J. 288, 56–80. 10.1111/febs.15570 32961620

[B32] Griffiths T. D. (1984). S-phase Transit Times as a Function of Age in Human Diploid Fibroblasts. Mech. Ageing Dev. 24, 273–282. 10.1016/0047-6374(84)90113-1 6717092

[B33] Grove G. L. Cristofalo V. J. (1977). Characterization of the Cell Cycle of Cultured Human Diploid Cells: Effects of Aging and Hydrocortisone. J. Cell. Physiol. 90, 415–422. 10.1002/jcp.1040900305 856836

[B34] Gruber H. E. Ingram J. A. Norton H. J. Hanley E. N. (2007). Senescence in Cells of the Aging and Degenerating Intervertebral Disc: Immunolocalization of Senescence-Associated Beta-Galactosidase in Human and Sand Rat Discs. Spine (Phila Pa 1976) 32, 321–327. 10.1097/01.brs.0000253960.57051.de 17268263

[B35] Hayflick L. (1965). The Limited *In Vitro* Lifetime of Human Diploid Cell Strains. Exp. Cell. Res. 37, 617–636. 10.1016/0014-4827(65)90211-9 14315085

[B36] Hayflick L. (1979). The Cell Biology of Aging. J. Invest. Dermatol. 73, 8–14. 10.1111/1523-1747.ep12532752 448179

[B37] Hayflick L. (1990). In the Interest of Clearer Communication. *In Vitro* Cell. Dev. Biol. 26, 1–2. 10.1007/bf02624146 2307636

[B38] Hayflick L. Moorhead P. S. (1961). The Serial Cultivation of Human Diploid Cell Strains. Exp. Cell Res. 25, 585–621. 10.1016/0014-4827(61)90192-6 13905658

[B39] Herbig U. Ferreira M. Condel L. Carey D. Sedivy J. M. (2006). Cellular Senescence in Aging Primates. Science 311, 1257. 10.1126/science.1122446 16456035

[B40] Hildebrand D. Lehle S. Borst A. Haferkamp S. Essmann F. Schulze-Osthoff K. (2013). α-Fucosidase as a Novel Convenient Biomarker for Cellular Senescence. Cell Cycle 12, 1922–1927. 10.4161/cc.24944 23673343 PMC3735706

[B41] Jeyapalan J. C. Ferreira M. Sedivy J. M. Herbig U. (2007). Accumulation of Senescent Cells in Mitotic Tissue of Aging Primates. Mech. Ageing Dev. 128, 36–44. 10.1016/j.mad.2006.11.008 17116315 PMC3654105

[B42] Ji G. Liu K. Okuka M. Liu N. Liu L. (2012). Association of Telomere Instability with Senescence of Porcine Cells. BMC Cel Biol 13, 36. 10.1186/1471-2121-13-36 PMC3563453 23241441

[B43] Jimenez A. G. Downs C. J. Lalwani S. Cipolli W. (2020). Cellular Metabolism and IL-6 Concentrations during Stimulated Inflammation in Small and Large Dog Breeds' Primary Fibroblasts Cells, as They Age. J. Exp. Biol. 224, jeb233734. 10.1242/jeb.233734 33443062

[B44] Jones R. B. Whitney R. G. Smith J. R. (1985). Intramitotic Variation in Proliferative Potential: Stochastic Events in Cellular Aging. Mech. Ageing Dev. 29, 143–149. 10.1016/0047-6374(85)90014-4 3974307

[B45] Joselow A. Lynn D. Terzian T. Box N. F. (2017). Senescence-Like Phenotypes in Human Nevi. Methods Mol. Biol. 1534, 175–184. 10.1007/978-1-4939-6670-7_17 27812879 PMC5125895

[B46] Kalashnik L. Bridgeman C. J. King A. R. Francis S. E. Mikhalovsky S. Wallis C. (2000). A Cell Kinetic Analysis of Human Umbilical Vein Endothelial Cells. Mech. Ageing Dev. 120, 23–32. 10.1016/s0047-6374(00)00179-2 11087901

[B47] Kill I. R. Faragher R. G. Lawrence K. Shall S. (1994). The Expression of Proliferation-dependent Antigens during the Lifespan of normal and Progeroid Human Fibroblasts in Culture. J. Cell Sci. 107, 571–579. 10.1242/jcs.107.2.571 7911472

[B48] Killilea D. W. Atamna H. Liao C. Ames B. N. (2003). Iron Accumulation during Cellular Senescence in Human FibroblastsIn Vitro. Antioxid. Redox Signaling 5, 507–516. 10.1089/152308603770310158 PMC4503765 14580305

[B49] Kim K.-W. Ha K.-Y. Lee J.-S. Na K.-H. Kim Y.-Y. Woo Y.-K. (2008). Senescence of Nucleus Pulposus Chondrocytes in Human Intervertebral Discs. Asian Spine J. 2, 1–8. 10.4184/asj.2008.2.1.1 20411135 PMC2857488

[B50] Kohler J. Popov C. Klotz B. Alberton P. Prall W. C. Haasters F. (2013). Uncovering the Cellular and Molecular Changes in Tendon Stem/progenitor Cells Attributed to Tendon Aging and Degeneration. Aging Cell 12, 988–999. 10.1111/acel.12124 23826660 PMC4225469

[B51] Kueper T. Grune T. Prahl S. Lenz H. Welge V. Biernoth T. (2007). Vimentin Is the Specific Target in Skin Glycation. J. Biol. Chem. 282, 23427–23436. 10.1074/jbc.m701586200 17567584

[B52] Kurz D. J. Decary S. Hong Y. Erusalimsky J. D. (2000). Senescence-associated (Beta)-galactosidase Reflects an Increase in Lysosomal Mass during Replicative Ageing of Human Endothelial Cells. J. Cell Sci. 113, 3613–3622. 10.1242/jcs.113.20.3613 11017877

[B53] Li Y. Yan Q. Wolf N. S. (1997). Long-term Caloric Restriction Delays Age-Related Decline in Proliferation Capacity of Murine Lens Epithelial Cells *In Vitro* and *In Vivo* . Invest. Ophthalmol. Vis. Sci. 38, 100–107. 9008635

[B54] Lipetz J. Cristofalo V. J. (1972). Ultrastructural Changes Accompanying the Aging of Human Diploid Cells in Culture. J. Ultrastruct. Res. 39, 43–56. 10.1016/s0022-5320(72)80005-4 5017037

[B55] Littlefield J. W. (1973). Attempted Hybridizations with Senescent Human Fibroblasts. J. Cell. Physiol. 82, 129–131. 10.1002/jcp.1040820115 4729512

[B56] Liu Y. Sanoff H. K. Cho H. Burd C. E. Torrice C. Ibrahim J. G. (2009). Expression ofp16INK4ain Peripheral Blood T-Cells Is a Biomarker of Human Aging. Aging Cell 8, 439–448. 10.1111/j.1474-9726.2009.00489.x 19485966 PMC2752333

[B57] Ma Y. Prigent S. A. Born T. L. Monell C. R. Feramisco J. R. Bertolaet B. L. (1999). Microinjection of Anti-p21 Antibodies Induces Senescent Hs68 Human Fibroblasts to Synthesize DNA but Not to divide. Cancer Res. 59, 5341–5348. 10537318

[B58] Macieira-Coelho A. Pontén J. Philipson L. (1966). The Division Cycle and RNA-Synthesis in Diploid Human Cells at Different Passage Levels *In Vitro* . Exp. Cel. Res. 42, 673–684. 10.1016/0014-4827(66)90280-1 5922078

[B59] Maeda K. Yasumoto S. Tsuruda A. Andoh K. Kai K. Otoi T. (2007). Establishment of a Novel Equine Cell Line for Isolation and Propagation of Equine Herpesviruses. J. Vet. Med. Sci. 69, 989–991. 10.1292/jvms.69.989 17917390

[B60] Masaldan S. Clatworthy S. A. S. Gamell C. Meggyesy P. M. Rigopoulos A.-T. Haupt S. (2018). Iron Accumulation in Senescent Cells Is Coupled with Impaired Ferritinophagy and Inhibition of Ferroptosis. Redox Biol. 14, 100–115. 10.1016/j.redox.2017.08.015 28888202 PMC5596264

[B61] McCormick J. J. Maher V. M. (1988). Towards an Understanding of the Malignant Transformation of Diploid Human Fibroblasts. Mutat. Res. 199, 273–291. 10.1016/0027-5107(88)90209-6 3287148

[B62] Meguro R. Asano Y. Odagiri S. Li C. Iwatsuki H. Shoumura K. (2007). Nonheme-iron Histochemistry for Light and Electron Microscopy: a Historical, Theoretical and Technical Review. Arch. Histology Cytol. 70, 1–19. 10.1679/aohc.70.1 17558140

[B63] Melk A. Kittikowit W. Sandhu I. Halloran K. M. Grimm P. Schmidt B. M. W. (2003). Cell Senescence in Rat Kidneys *In Vivo* Increases with Growth and Age Despite Lack of Telomere Shortening. Kidney Int. 63, 2134–2143. 10.1046/j.1523-1755.2003.00032.x 12753300

[B64] Montpetit A. J. Alhareeri A. A. Montpetit M. Starkweather A. R. Elmore L. W. Filler K. (2014). Telomere Length. Nurs. Res. 63, 289–299. 10.1097/nnr.0000000000000037 24977726 PMC4292845

[B65] Moreno-García A. Kun A. Calero O. Medina M. Calero M. (2018). An Overview of the Role of Lipofuscin in Age-Related Neurodegeneration. Front. Neurosci. 1, 1–13. 10.3389/fnins.2018.00464 PMC6041410 30026686

[B66] Nelson D. M. McBryan T. Jeyapalan J. C. Sedivy J. M. Adams P. D. (2014). A Comparison of Oncogene-Induced Senescence and Replicative Senescence: Implications for Tumor Suppression and Aging. Age (Dordr) 36, 9637. 10.1007/s11357-014-9637-0 24647599 PMC4082585

[B67] Noda A. Ning Y. Venable S. F. Pereira-Smith O. M. Smith J. R. (1994). Cloning of Senescent Cell-Derived Inhibitors of DNA Synthesis Using an Expression Screen. Exp. Cel. Res. 211, 90–98. 10.1006/excr.1994.1063 8125163

[B68] Noppe G. Dekker P. de Koning-Treurniet C. Blom J. van Heemst D. Dirks R. W. (2009). Rapid Flow Cytometric Method for Measuring Senescence Associated β-galactosidase Activity in Human Fibroblasts. Cytometry 75A, 910–916. 10.1002/cyto.a.20796 19777541

[B69] Norsgaard H. Clark B. F. C. Rattan S. I. S. (1996). Distinction between Differentiation and Senescence and the Absence of Increased Apoptosis in Human Keratinocytes Undergoing Cellular Aging *In Vitro* . Exp. Gerontol. 31, 563–570. 10.1016/0531-5565(96)00011-3 9415111

[B70] Norwood T. H. Smith J. R. Stein G. H. (1990). “Aging at the Cellular Level: The Human Fibroblastlike Cell Model,” in Handbook of the Biology of Aging. Editors Schneider EL Rowe JW. Third Edition (New York Academic Press).

[B71] Oh H.-Y. Jin X. Kim J.-G. Oh M.-J. Pian X. Kim J.-M. (2007). Characteristics of Primary and Immortalized Fibroblast Cells Derived from the Miniature and Domestic Pigs. BMC Cell Biol. 8, 20. 10.1186/1471-2121-8-20 17543094 PMC1894962

[B72] Okamoto Y. Monjushiro H. Fukumoto T. Watarai H. (2006). Measurement of Hydrolysis Kinetics of Galactose-Substituted Fluorescein by β-galactosidase at the Toluene-Water Interface by Spinning Microtube Fluorometry. Anal. Bioanal. Chem. 385, 1430–1438. 10.1007/s00216-006-0551-x 16838159

[B73] Olovnikov A. M. (1973). A Theory of Marginotomy. J. Theor. Biol. 41, 181–190. 10.1016/0022-5193(73)90198-7 4754905

[B74] Overall A. D. Faragher R. G. (2019). Population Type Influences the Rate of Ageing. Heredity 123, 273–282. 10.1038/s41437-019-0187-1 30737473 PMC6781125

[B75] Paradis V. Youssef N. Dargère D. Bâ N. Bonvoust F. Deschatrette J. (2001). Replicative Senescence in normal Liver, Chronic Hepatitis C, and Hepatocellular Carcinomas. Hum. Pathol. 32, 327–332. 10.1053/hupa.2001.22747 11274643

[B76] Perillo N. L. Walford R. L. Newman M. A. Effros R. B. (1989). Human T Lymphocytes Possess a Limited *In Vitro* Life Span. Exp. Gerontol. 24, 177–187. 10.1016/0531-5565(89)90009-0 2786475

[B77] Pontén J. Stein W. D. Shall S. (1983). A Quantitative Analysis of the Aging of Human Glial Cells in Culture. J. Cell. Physiol. 117, 342–352. 10.1002/jcp.1041170309 6361044

[B78] Poot M. Visser W. J. Verkerk A. Jongkind J. F. (1985). Autofluorescence of Human Skin Fibroblasts during Growth Inhibition and *In Vitro* Ageing. Gerontology 31 (3), 158–165. 10.1159/000212697 4018588

[B79] Porter M. B. Pereira-Smith O. M. Smith J. R. (1992). Common Senescent Cell-specific Antibody Epitopes on Fibronectin in Species and Cells of Varied Origin. J. Cell. Physiol. 150, 545–551. 10.1002/jcp.1041500315 1371514

[B80] Porter M. B. Pereira-Smith O. M. Smith J. R. (1990). Novel Monoclonal Antibodies Identify Antigenic Determinants Unique to Cellular Senescence. J. Cell. Physiol. 142, 425–433. 10.1002/jcp.1041420228 1689321

[B81] Rawes V. Kipling D. Kill I. R. Faragher R. G. (1997). The Kinetics of Senescence in Retinal Pigmented Epithelial Cells: a Test for the Telomere Hypothesis of Ageing?. Biochemistry (Mosc) 62, 1291–1295. 9467853

[B82] Röhme D. (1981). Evidence for a Relationship between Longevity of Mammalian Species and Life Spans of normal Fibroblasts *In Vitro* and Erythrocytes *In Vivo* . Proc. Natl. Acad. Sci. 78, 5009–5013. 10.1073/pnas.78.8.5009 6946449 PMC320321

[B83] Safir Filho M. Dao P. Gesson M. Martin A. R. Benhida R. (2018). Development of Highly Sensitive Fluorescent Probes for the Detection of β-galactosidase Activity - Application to the Real-Time Monitoring of Senescence in Live Cells. Analyst 143, 2680–2688. 10.1039/c8an00516h 29774897

[B84] Sajithlal G. B. Gowri Chandrakasan G. (1999). Role of Lipid Peroxidation Products in the Formation of Advanced Glycation End Products: An *In Vitro* Study on Collagen. Proc. Indian Acad. Sci. - Chem. Sci. 111, 215–229.

[B85] Sándor S. Kubinyi E. (2019). Genetic Pathways of Aging and Their Relevance in the Dog as a Natural Model of Human Aging. Front. Genet. 10, 948. 10.3389/fgene.2019.00948 31681409 PMC6813227

[B86] Schafer M. J. White T. A. Evans G. Tonne J. M. Verzosa G. C. Stout M. B. (2016). Exercise Prevents Diet-Induced Cellular Senescence in Adipose Tissue. Diabetes 65, 1606–1615. 10.2337/db15-0291 26983960 PMC4878429

[B87] Schneider E. L. Mitsui Y. (1976). The Relationship between *In Vitro* Cellular Aging and *In Vivo* Human Age. Proc. Natl. Acad. Sci. 73, 3584–3588. 10.1073/pnas.73.10.3584 1068470 PMC431162

[B88] Sell D. R. Primc M. Schafer I. A. Kovach M. Weiss M. A. Monnier V. M. (1998). Cell-associated Pentosidine as a Marker of Aging in Human Diploid Cells *In Vitro* and *In Vivo* . Mech. Ageing Development 105, 221–240. 10.1016/s0047-6374(98)00090-6 9862232

[B89] Severino J. Allen R. G. Balin S. Balin A. Cristofalo V. J. (2000). Is β-Galactosidase Staining a Marker of Senescence *In Vitro* and *In Vivo*?. Exp. Cel. Res. 257, 162–171. 10.1006/excr.2000.4875 10854064

[B90] Shall S. (1987). “Mortalisation or Reproductive Sterility of Mammalian Cells in Culture,” in Perspectives on Mammalian Cell Death. Editor Potten CS (Oxford: Oxford University Press), 184–201.

[B91] Shay J. W. Van Der Haegen B. A. Ying Y. Wright W. E. (1993). The Frequency of Immortalization of Human Fibroblasts and Mammary Epithelial Cells Transfected with SV40 Large T-Antigen. Exp. Cell Res. 209, 45–52. 10.1006/excr.1993.1283 8224005

[B92] Sherwood S. W. Rush D. Ellsworth J. L. Schimke R. T. (1988). Defining Cellular Senescence in IMR-90 Cells: a Flow Cytometric Analysis. Proc. Natl. Acad. Sci. 85, 9086–9090. 10.1073/pnas.85.23.9086 3194411 PMC282668

[B93] Shi J. Pang L. Jiao S. (2018). The Response of Nucleus Pulposus Cell Senescence to Static and Dynamic Compressions in a Disc Organ Culture. Biosci. Rep. 38, BSR20180064. 10.1042/bsr20180064 29437905 PMC5843747

[B95] Smith J. R. Hayflick L. (1974). Variation in the Life-Span of Clones Derived from Human Diploid Cell Strains. J. Cel Biol 62, 48–53. 10.1083/jcb.62.1.48 PMC2109186 4407044

[B96] Smith J. R. Whitney R. (1980). Intraclonal Variation in Proliferative Potential of Human Diploid Fibroblasts: Stochastic Mechanism for Cellular Aging. Science 207, 82–84. 10.1126/science.7350644 7350644

[B97] Stanulis-Praeger B. (1987). Cellular Senescence Revisited: a Review. Mech. Ageing Development 38, 1–48. 10.1016/0047-6374(87)90109-6 2439851

[B98] Stein G. H. Drullinger L. F. Soulard A. Dulić V. (1999). Differential Roles for Cyclin-dependent Kinase Inhibitors P21 and P16 in the Mechanisms of Senescence and Differentiation in Human Fibroblasts. Mol. Cell. Biol. 19, 2109–2117. 10.1128/mcb.19.3.2109 10022898 PMC84004

[B99] Thomas E. al-Baker E. Dropcova S. Denyer S. Ostad N. Lloyd A. (1997). Different Kinetics of Senescence in Human Fibroblasts and Peritoneal Mesothelial Cells. Exp. Cell Res. 236, 355–358. 10.1006/excr.1997.3760 9344618

[B110] van Duijn S. Nabuurs R. J. A. van Duinen S. G. Natté R. (2013). Comparison of Histological Techniques to Visualize Iron in Paraffin-Embedded Brain Tissue of Patients With Alzheimer's Disease. J. Histochem. Cytochem. 61, 785–792. 10.1369/0022155413501325 23887894 PMC3808575

[B100] Waaijer M. E. C. Croco E. Westendorp R. G. J. Slagboom P. E. Sedivy J. M. Lorenzini A. (2016). DNA Damage Markers in Dermal Fibroblasts *In Vitro* Reflect Chronological Donor Age. Aging 8, 147–155. 10.18632/aging.100890 26830451 PMC4761719

[B101] Waaijer M. E. C. Parish W. E. Strongitharm B. H. van Heemst D. Slagboom P. E. de Craen A. J. M. (2012). The Number of p16INK4a Positive Cells in Human Skin Reflects Biological Age. Aging Cell 11, 722–725. 10.1111/j.1474-9726.2012.00837.x 22612594 PMC3539756

[B102] Weismann A. (1889). Essays upon Heredity and kindred Biological Problems. Oxford: Clarendon Press. 10.5962/bhl.title.11364

[B103] Wyllie F. S. Jones C. J. Skinner J. W. Haughton M. F. Wallis C. Wynford-Thomas D. (2000). Telomerase Prevents the Accelerated Cell Ageing of Werner Syndrome Fibroblasts. Nat. Genet. 24, 16–17. 10.1038/71630 10615119

[B104] Xia M. L. Xie X. H. Ding J. H. Du R. H. Hu G. (2020). Astragaloside IV Inhibits Astrocyte Senescence: Implication in Parkinson's Disease. J. Neuroinflammation 17, 105. 10.1186/s12974-020-01791-8 32252767 PMC7137443

[B105] Yang J. Y. Park M. J. Park S. Lee E.-S. (2018). Increased Senescent CD8+ T Cells in the Peripheral Blood Mononuclear Cells of Behçet's Disease Patients. Arch. Dermatol. Res. 310, 127–138. 10.1007/s00403-017-1802-8 29255925

[B106] Yin D. (1996). Biochemical Basis of Lipofuscin, Ceroid, and Age Pigment-like Fluorophores. Free Radic. Biol. Med. 21, 871–888. 10.1016/0891-5849(96)00175-x 8902532

[B107] You S. Moon J.-H. Kim T.-K. Kim S.-C. Kim J.-W. Yoon D.-H. (2004). Cellular Characteristics of Primary and Immortal Canine Embryonic Fibroblast Cells. Exp. Mol. Med. 36, 325–335. 10.1038/emm.2004.43 15365251

[B108] Zou Y. Misri S. Shay J. W. Pandita T. K. Wright W. E. (2009). Altered States of Telomere Deprotection and the Two-Stage Mechanism of Replicative Aging. Mcb 29, 2390–2397. 10.1128/mcb.01569-08 19223460 PMC2668373

